# Data survey of students behavioral and psychological adaptations in disaster-prone areas of Mount Merapi in Indonesia

**DOI:** 10.1016/j.dib.2022.108229

**Published:** 2022-04-29

**Authors:** Siti Hadiyati Nur Hafida, Nor Kalsum Mohd Isa, Moh Hairy Ibrahim, Jumadi, Muhamad Toyib, Muhammad Musiyam

**Affiliations:** a Department of Geography Education, Faculty of Teacher Training and Education, Universitas Muhammadiyah Surakarta, Indonesia; b Department of Geography, Faculty of Human Science, Universiti Pendidikan Sultan Idris, Malaysia; c Department of Geography, Faculty of Geography, Universitas Muhammadiyah Surakarta, Indonesia; d Department of Mathematic Education, Faculty of Teacher Training and Education, Universitas Muhammadiyah Surakarta, Indonesia

## Abstract

This set of data provides the data related to the measurement of behavioral and psychological adaptation on students in disaster-prone areas of Mount Merapi in Indonesia. The survey was conducted online by considering respondents agreement in filling the demography data (4 questions), behavioral adaptation (17 questions) and psychological adaptation (9 questions). The survey was done on March–June 2021. Total respondents were 364 students who were selected through probability-based cluster sampling for schools around disaster-prone areas 1–3 of Mount Merapi eruption. Respondents were from 15 private and state schools at the slope of Mount Merapi (Central Java and Yogyakarta, Indonesia) and categorized as disaster-prone areas of eruption. The survey data were analyzed using multivariate analysis. The data can help educators, researchers or the government to find out students’ preparedness level in dealing with Mount Merapi eruption in the future and to strengthen the integration of educational management disasters in the school curriculum.

## Specification Table

**Table:** 

Subject	Social sciences
Specific subject area	Educational management Disaster at school
Type of data	Primary data, Tables
How data were acquired	Data were acquired by online survey (Google Form) to students from selected schools. The data then were input into Excel and analyzed using SPSS software.
Data format	Raw, analyzed, and descriptive data
Parameters for data collection	The survey data were collected from 364 students from March to June 2021.
Description of data collection	Probability sampling and cluster sampling were used to decide the respondents through Google Form that were given to 15 schools at the slope of Mount Merapi, Indonesia.
Data source location	Survey was conducted in 15 schools in disaster-prone areas 1–3 of Mount Merapi, Indonesia.
Data accessibility	Dataset is uploaded on Mendeley Repository name: Mendeley Data identification number: 10.17632/sghk2hgdmb.4 Direct URL to data: https://data.mendeley.com/datasets/sghk2hgdmb/4

## Values of the Data

• Dataset helps to acquire empiric proofs of students’ preparedness in dealing with the eruption of Mount Merapi based on their adaptation skills, both behavioral and psychological.
• Dataset helps researcher who wants to compare it with other disaster-related researches with the purpose to help students strengthen their endurance in dealing with Mount Merapi eruption.
• Dataset may help educators and the government to strengthen the integration of educational management disasters in the Indonesian curriculum to create strong generations.

## 1 Data Description

Data in this article informs about the students’ level of preparedness in dealing with disaster which is measured through the students’ behavioral and psychological adaptation. The survey involved 364 students from 15 different schools in disaster-prone areas (KRB) 1–3 of Mount Merapi. The focus of the data collection consists of three variables; (A) Demography, including name, gender, class, and schools. (B) The variables of behavioral adaptation (PR) include disaster understanding, disaster preparedness, network, disaster communication, participation and policy. The indicators are in the form of 17 questions related to behavioral adaptation. (C) Nine questions related to psychological adaptation (PS) are based on seven indicators such as self-confidence, courage, positive feelings, self-composure, social support, anxiety and stress, and mental readiness. Questionnaire is provided as additional data, while the results of data based on three variables can be seen in the student sheet in the Mendeley section.

All the questions used to measure the variables of behavioral and psychological adaptations in this form are by demography data by a Likert scale of 5 interval point (Strongly Disagree to Strongly Agree) which can be seen in Table 1–6. Table 1 shows the respondents characteristics based on the demography and social economy of the respondents. Table 2 shows the frequency of distribution on behavioral and psychological adaptation question. Tables 3 and 4 show the relations between the demographic characteristics of respondents and the variables of students’ behavioral and psychological adaptation in disaster-prone areas of Mount Merapi eruption. Table 5 describes the frequency distribution data for each respondent's answer to the existing question, while Table 6 shows the frequency distribution data and the correlation between the demographic characteristics of the respondents with behavioral and psychological adaptation variables.

**Table 1:** Demographic characteristics of respondents.

Variable	Category	Frequency	Percentage	Range	Min	Max
Gender	Male	140	38.46	1	1	2
	Female	224	61.54			

Age	16 years	36	9.89	4	1	5
	17 years	132	36.26			
	18 years	143	39.29			
	19 years	39	10.71			
	20 years	14	3.85			

KRB	KRB 1	258	70.88	2	1	3
	KRB 2	57	15.66			
	KRB 3	49	13.46			

Class	X	123	33.79	2	1	3
	XI	152	41.76			
	XII	89	24.45

**Table 2:** Descriptive statistics on behavioral adaptation and psychological adaptation (*n* = 364).

		Mean		95% confidence interval for Mean							Skewness		Kurtosis	
Variable	Item	Statistik	Std error	Lower bound	Upper bound	Var	Std Dev	Min	Max	Range	Stat	Std error	Stat	Std error
Behavioral Adaptation	I have understood the potential disasters around my school	3.8626	0.0516	3.7613	3.9640	0.9673	0.9835	1.0000	5.0000	4.0000	−0.5251	0.1279	−0.3579	0.2550
	I always find out information about the condition of Mount Merapi	3.6676	0.0568	3.5558	3.7793	1.1757	1.0843	1.0000	5.0000	4.0000	−0.2999	0.1279	−0.7960	0.2550
	I am familiar with the signs of the eruption of Mount Merapi	3.9176	0.0525	3.8144	4.0207	1.0015	1.0007	1.0000	5.0000	4.0000	−0.6631	0.1279	−0.1204	0.2550
	I know the impact caused by the eruption of Mount Merapi	4.2637	0.0490	4.1675	4.3600	0.8724	0.9340	1.0000	5.0000	4.0000	−1.3232	0.1279	1.5481	0.2550
	I have prepared myself to anticipate the occurrence of the eruption of Mount Merapi	3.8187	0.0506	3.7192	3.9181	0.9312	0.9650	1.0000	5.0000	4.0000	−0.4437	0.1279	−0.3555	0.2550
	I know what items must be prepared in case of a catastrophic emergency	3.9753	0.0542	3.8686	4.0819	1.0710	1.0349	1.0000	5.0000	4.0000	−0.8051	0.1279	−0.0175	0.2550
	I realize that disaster adaptation is able to minimize disaster victims	4.3434	0.0457	4.2535	4.4333	0.7605	0.8721	1.0000	5.0000	4.0000	−1.3288	0.1279	1.5248	0.2550
	I know what to do when an eruption strikes	4.1291	0.0487	4.0334	4.2248	0.8621	0.9285	1.0000	5.0000	4.0000	−0.9448	0.1279	0.5349	0.2550
	I have used communication technology (such as: TV, radio) to improve disaster adaptability	4.1044	0.0523	4.0015	4.2073	0.9973	0.9987	1.0000	5.0000	4.0000	−0.9450	0.1279	0.2981	0.2550
	I can easily use the materials around me to be used as disaster warnings	3.6181	0.0570	3.5060	3.7303	1.1844	1.0883	1.0000	5.0000	4.0000	−0.3443	0.1279	−0.6082	0.2550
	I have established relationships with people or groups outside of the eruption prone areas to understand their disaster preparedness management	3.4093	0.0651	3.2813	3.5374	1.5427	1.2421	1.0000	5.0000	4.0000	−0.2250	0.1279	−0.9742	0.2550
	I realize that communication is an important factor that influencing disaster adaptation patterns	4.2720	0.0470	4.1795	4.3644	0.8046	0.8970	1.0000	5.0000	4.0000	−1.1155	0.1279	0.7109	0.2550
	I quickly found out emergency contact information that could be contacted during a disaster situation	3.6044	0.0582	3.4900	3.7188	1.2315	1.1097	1.0000	5.0000	4.0000	−0.3505	0.1279	−0.7097	0.2550
	I feel responsible for being actively involved in increasing the adaptive capacity at school	3.6593	0.0529	3.5553	3.7634	1.0186	1.0093	1.0000	5.0000	4.0000	−0.3280	0.1279	−0.4451	0.2550
	I believe that every policy, program or facility provided by schools can be used to improve disaster adaptation	4.0000	0.0516	3.8985	4.1015	0.9697	0.9847	1.0000	5.0000	4.0000	−0.7136	0.1279	−0.0822	0.2550
	I am open to new ideas to support increasing the capacity of disaster adaptation in schools	3.8462	0.0544	3.7391	3.9532	1.0782	1.0384	1.0000	5.0000	4.0000	−0.5197	0.1279	−0.4833	0.2550
	I can adapt in facing future disaster challenges	3.8489	0.0511	3.7485	3.9493	0.9496	0.9745	1.0000	5.0000	4.0000	−0.6092	0.1279	0.0396	0.2550
Psychological Adaptation	I feel confident in my ability to deal with disaster situations	3.6758	0.0526	3.5724	3.7793	1.0076	1.0038	1.0000	5.0000	4.0000	−0.3832	0.1279	−0.3104	0.2550
	I do not feel afraid when facing the catastrophic eruption of Mount Merapi	3.5082	0.0588	3.3926	3.6239	1.2589	1.1220	1.0000	5.0000	4.0000	−0.3913	0.1279	−0.4657	0.2550
	I can maintain a good feeling in a disaster situation	3.7747	0.0525	3.6716	3.8779	1.0015	1.0007	1.0000	5.0000	4.0000	−0.4980	0.1279	−0.2434	0.2550
	I can stay calm when in a disaster emergency situation	3.5934	0.0606	3.4742	3.7126	1.3384	1.1569	1.0000	5.0000	4.0000	−0.4704	0.1279	−0.5693	0.2550
	I know strategies that I can use to calm down when a disaster situation	3.8159	0.0535	3.7107	3.9212	1.0432	1.0214	1.0000	5.0000	4.0000	−0.4670	0.1279	−0.5198	0.2550
	If I am in the situation of a volcano eruption disaster, I know how to respond to that situation	3.8214	0.0498	3.7235	3.9193	0.9019	0.9497	1.0000	5.0000	4.0000	−0.4319	0.1279	−0.2959	0.2550
	If people around me experience stress due to disasters, I can calm them down	3.4093	0.0584	3.2946	3.5241	1.2397	1.1134	1.0000	5.0000	4.0000	−0.1376	0.1279	−0.7202	0.2550
	I can realize when I experience anxiety and stress due to disasters	3.5247	0.0584	3.4099	3.6396	1.2418	1.1144	1.0000	5.0000	4.0000	−0.2786	0.1279	−0.6732	0.2550
	I have prepared mentally for the worst situation that I will experience during a disaster	3.8297	0.0514	3.7285	3.9308	0.9626	0.9811	1.0000	5.0000	4.0000	−0.4623	0.1279	−0.3428	0.2550

**Table 3:** Descriptive statistics on demographic characteristics of respondents' and behavioral adaptation (PR).

		Psychological Adaptation (PR)																
Variable	Category	PR1	PR2	PR3	PR4	PR5	PR6	PR7	PR8	PR9	PR10	PR11	PR12	PR13	PR14	PR15	PR16	PR17
Gender	Male	3.8214	3.7643	3.9286	4.2643	3.9143	3.9500	4.2929	4.2286	4.0357	3.7500	3.5643	4.1143	3.6643	3.5500	3.9714	3.8357	3.8357
	Female	3.8884	3.6071	3.9107	4.2634	3.7589	3.9911	4.3750	4.0670	4.1473	3.5357	3.3125	4.3705	3.5670	3.7277	4.0179	3.8527	3.8571
	F test	0.3987	1.8136	0.0274	0.0001	2.2406	0.1354	0.7639	2.6217	1.0762	3.3620	3.5654	7.1501	0.6620	2.6825	0.1911	0.0229	0.0416
	Significant	0.5281	0.1789	0.8687	0.9929	0.1353	0.7131	0.3827	0.1063	0.3002	0.0675	0.0598	0.0078	0.4164	0.1023	0.6623	0.8797	0.8386

Age	16 years	3.7778	3.6111	3.8611	4.3611	3.7500	4.0278	4.3333	4.2500	4.1389	3.5556	3.1389	4.4722	3.3056	3.5000	4.2778	3.6111	3.7778
	17 years	3.8106	3.5379	3.8788	4.3106	3.7955	3.9621	4.3561	4.1667	4.0833	3.5530	3.2955	4.2045	3.4545	3.6212	3.9621	3.7879	3.8106
	18 years	3.8671	3.6923	3.9301	4.3007	3.8462	4.0280	4.3357	4.1399	4.1748	3.6643	3.5105	4.3007	3.7133	3.6084	3.9510	3.9301	3.8741
	19 years	3.9744	3.8974	4.2051	3.8718	3.7949	3.7692	4.3590	3.8718	3.8718	3.7692	3.6667	4.2821	3.7949	4.0769	4.0513	3.8718	3.9744
	20 years	4.2143	4.1429	3.5000	4.2857	4.0000	4.0000	4.2857	4.0714	4.1429	3.5000	3.4286	4.0714	4.1429	3.7857	4.0000	4.0714	3.7857
	F test	0.7312	1.6372	1.5066	1.9767	0.2213	0.5069	0.0291	0.9734	0.7352	0.4387	1.3661	0.8464	2.7625	2.1121	0.8786	0.9689	0.2969
	Significant	0.5711	0.1643	0.1997	0.0975	0.9265	0.7307	0.9984	0.4220	0.5684	0.7806	0.2452	0.4965	0.0276	0.0788	0.4768	0.4245	0.8799

KRB	KRB 1	3.7558	3.6395	3.8953	4.2519	3.7907	3.9225	4.3256	4.0853	4.0775	3.6047	3.3643	4.2287	3.6124	3.6783	3.9535	3.8566	3.8217
	KRB 2	4.0702	3.4561	3.8772	4.2632	3.7018	3.9123	4.3684	4.1053	3.9649	3.5263	3.4386	4.2456	3.3333	3.4737	4.0000	3.7193	3.8246
	KRB 3	4.1837	4.0612	4.0816	4.3265	4.1020	4.3265	4.4082	4.3878	4.4082	3.7959	3.6122	4.5306	3.8776	3.7755	4.2449	3.9388	4.0204
	F test	5.5363	4.4836	0.7675	0.1307	2.6636	3.3052	0.2115	2.2225	2.9475	0.8759	0.8382	2.3799	3.2307	1.3370	1.8112	0.6321	0.8766
	Significant	0.0043	0.0119	0.4649	0.8775	0.0711	0.0378	0.8095	0.1098	0.0537	0.4174	0.4333	0.0940	0.0407	0.2639	0.1649	0.5321	0.4171

Class	X	3.8618	3.6911	3.8699	4.2846	3.9350	4.2927	4.2927	4.1626	4.1951	3.7317	3.4553	4.2358	3.4959	3.6748	4.1301	3.7642	3.8293
	XI	3.8553	3.6513	3.9145	4.2303	3.7368	4.3421	4.3421	4.0921	4.0658	3.5329	3.3487	4.2303	3.6184	3.6184	3.8750	3.8224	3.7829
	XII	3.8764	3.6629	3.9888	4.2921	3.7978	4.4157	4.4157	4.1461	4.0449	3.6067	3.4494	4.3933	3.7303	3.7079	4.0337	4.0000	3.9888
	F test	0.0130	0.0465	0.3641	0.1685	1.4642	1.0509	0.5129	0.2147	0.7779	1.1418	0.3106	1.0785	1.1739	0.2412	2.3676	1.4027	1.2925
	Significant	0.9871	0.9546	0.6951	0.8450	0.2326	0.3507	0.5992	0.8069	0.4601	0.3204	0.7332	0.3412	0.3103	0.7858	0.0952	0.2473	0.2759

**Table 4:** Descriptive statistics on demographic characteristics of respondents' and psychological adaptation (PS).

		Psychological Adaptation (PS)								
Variable	Category	PS1	PS2	PS3	PS4	PS5	PS6	PS7	PS8	PS9
Gender	Male	3.7286	3.6214	3.8786	3.7429	3.9286	3.8643	3.3214	3.3714	3.9429
	Female	3.6429	3.4375	3.7098	3.5000	3.7455	3.7946	3.4643	3.6205	3.7589
	F test	0.6276	2.3236	2.4597	3.8262	2.7805	0.4626	1.4199	4.3449	3.0447
	Significant	0.4288	0.1283	0.1177	0.0512	0.0963	0.4968	0.2342	0.0378	0.0818

Age	16 years	3.4722	3.4167	3.6944	3.4444	3.7778	3.6944	3.0556	3.6111	3.7778
	17 years	3.6894	3.4470	3.7273	3.5985	3.7955	3.7879	3.4621	3.4242	3.8106
	18 years	3.6224	3.4895	3.7483	3.5245	3.7343	3.8322	3.3916	3.6014	3.8042
	19 years	4.0000	3.8205	4.0256	3.7949	4.1282	3.9487	3.4872	3.4103	3.9487
	20 years	3.7143	3.6429	4.0000	4.0714	4.0714	4.0000	3.7857	3.7857	4.0714
	F test	1.5081	0.9737	0.9469	1.1723	1.3908	0.5028	1.4466	0.7846	0.4151
	Significant	0.1992	0.4219	0.4368	0.3227	0.2366	0.7337	0.2181	0.5357	0.7978

KRB	KRB 1	3.6395	3.5078	3.7558	3.5853	3.8256	3.8101	3.4612	3.5349	3.8605
	KRB 2	3.7544	3.5439	3.6491	3.5439	3.7193	3.7368	3.1404	3.4912	3.6491
	KRB 3	3.7755	3.4694	4.0204	3.6939	3.8776	3.9796	3.4490	3.5102	3.8776
	F test	0.5835	0.0578	1.9824	0.2424	0.3545	0.9237	1.9855	0.0404	1.1515
	Significant	0.5585	0.9438	0.1392	0.7848	0.7017	0.3980	0.1388	0.9604	0.3173

Class	X	3.6341	3.6423	3.8943	3.7398	3.9187	3.8374	3.4146	3.3902	3.9512
	XI	3.6776	3.3026	3.6711	3.4737	3.7237	3.7763	3.3947	3.6382	3.7171
	XII	3.7303	3.6742	3.7865	3.5955	3.8315	3.8764	3.4270	3.5169	3.8539
	F test	0.2365	4.4875	1.7067	1.8074	1.2547	0.3368	0.0255	1.6918	1.9821
	Significant	0.7895	0.0119	0.1829	0.1656	0.2864	0.7143	0.9748	0.1856	0.1393

**Table 5:** Frequency distribution of respondent responses to each item of indicator (*n* = 364).

	Strongly Agree		Agree		Neutral		Disagree		Sytrongly Disagree		
Indicators	F	%	F	%	F	%	F	%	F	%	Average
PR1	114	31%	114	34%	94	26%	26	7%	5	1%	3.8626
PR2	105	29%	105	25%	113	31%	43	12%	8	2%	3.6676
PR3	125	34%	125	32%	90	25%	21	6%	7	2%	3.9176
PR4	186	51%	186	30%	49	13%	10	3%	7	2%	4.2637
PR5	104	29%	104	34%	105	29%	24	7%	5	1%	3.8187
PR6	140	38%	140	31%	75	21%	25	7%	8	2%	3.9753
PR7	200	55%	200	28%	48	13%	8	2%	4	1%	4.3434
PR8	154	42%	154	34%	66	18%	13	4%	5	1%	4.1291
PR9	165	45%	165	28%	73	20%	16	4%	7	2%	4.1044
PR10	95	26%	95	27%	115	32%	41	11%	12	3%	3.6181
PR11	93	26%	93	21%	102	28%	64	18%	26	7%	3.4093
PR12	186	51%	186	29%	56	15%	12	3%	3	1%	4.2720
PR13	95	26%	95	28%	104	29%	49	13%	12	3%	3.6044
PR14	87	24%	87	31%	120	33%	33	9%	8	2%	3.6593
PR15	139	38%	139	30%	87	24%	17	5%	6	2%	4.0000
PR16	120	33%	120	29%	101	28%	28	8%	7	2%	3.8462
PR17	106	29%	106	36%	97	27%	19	5%	8	2%	3.8489
PS1	87	24%	87	32%	118	32%	29	8%	9	2%	3.6758
PS2	81	22%	81	28%	118	32%	39	11%	21	6%	3.5082
PS3	100	27%	100	34%	105	29%	26	7%	8	2%	3.7747
PS4	97	27%	97	28%	99	27%	42	12%	20	5%	3.5934
PS5	113	31%	113	30%	101	28%	30	8%	6	2%	3.8159
PS6	102	28%	102	35%	107	29%	21	6%	5	1%	3.8214
PS7	75	21%	75	24%	128	35%	57	16%	16	4%	3.4093
PS8	87	24%	87	26%	117	32%	48	13%	15	4%	3.5247
PS9	109	30%	109	32%	107	29%	22	6%	6	2%	3.8297

**Table 6:** Frequency distribution and correlation between the demographic characteristic respondents' and behavioral and psychological adaptation variables.

Characteristics	Sub- characteristics	Variable	Strongly Agree	Agree	Neutral	Disagree	Strongly Disagree	N	Max Score	Score	Mean	%	Category
Gender	Male	Behavioral adaptation	927	628	590	156	79	2380	11,900	9308	3.91092437	78%	High
		Psychological adaptation	379	340	389	102	50	1260	6300	4676	3.71111111	74%	High
	Female	Behavioral adaptation	1299	1231	924	295	59	3808	19,040	14,840	3.89705882	78%	High
		Psychological adaptation	473	649	626	212	56	2016	10,080	7319	3.63045635	73%	High

Age	16 years	Behavioral adaptation	1096	749	719	196	79	2839	14,195	11,104	3.91123635	78%	High
		Psychological adaptation	433	424	456	134	56	1503	7515	5553	3.69461078	74%	High
	17 years	Behavioral adaptation	1472	1184	994	280	99	4029	20,145	15,737	3.90593199	78%	High
		Psychological adaptation	587	642	620	209	75	2133	10,665	7856	3.68307548	74%	High
	18 years	Behavioral adaptation	1707	1439	1168	325	87	4726	23,630	18,532	3.9212865	78%	High
		Psychological adaptation	645	769	761	240	87	2502	12,510	9151	3.65747402	73%	High
	19 years	Behavioral adaptation	1484	1309	1048	309	66	4216	21,080	16,484	3.90986717	78%	High
		Psychological adaptation	556	701	692	223	60	2232	11,160	8166	3.65860215	73%	High
	20 years	Behavioral adaptation	1367	1265	966	302	61	3961	19,805	15,458	3.90254986	78%	High
		Psychological adaptation	502	677	646	215	57	2097	10,485	7643	3.64473057	73%	High
KRB	KRB 1	Behavioral adaptation	1537	1321	1091	314	123	4386	21,930	16,993	3.87437301	77%	High
		Psychological adaptation	607	692	738	207	78	2322	11,610	8509	3.66451335	73%	High
	KRB 2	Behavioral adaptation	267	368	251	78	5	969	4845	3721	3.84004128	77%	High
		Psychological adaptation	96	192	149	66	10	513	2565	1837	3.58089669	72%	High
	KRB 3	Behavioral adaptation	422	170	172	59	10	833	4165	3434	4.12244898	82%	Very high
		Psychological adaptation	149	105	128	41	18	441	2205	1649	3.73922902	75%	High

Class	X	Behavioral adaptation	1596	1440	1192	341	123	4692	23,460	18,121	3.86210571	77%	High
		Psychological adaptation	629	758	780	236	81	2484	12,420	9070	3.65136876	73%	High
	XI	Behavioral adaptation	928	1092	912	254	78	3264	16,320	12,330	3.77757353	76%	High
		Psychological adaptation	347	546	588	194	53	1728	8640	6124	3.54398148	71%	High
	XII	Behavioral adaptation	962	819	702	203	34	2720	13,600	10,632	3.90882353	78%	High
		Psychological adaptation	355	436	463	146	40	1440	7200	5240	3.63888889	73%	High

The data on Mendeley Data contains a questionnaire and a recapitulation of respondents’ answers based on behavioral and psychological adaptation questionnaires in disaster-prone areas of Mount Merapi eruption.

## 2 Experimental Design, Materials and Methods

The context of this research is high school students’ adaptation skills. Research respondents come from 15 schools around disaster-prone areas 1–3 of the eruption of Mount Merapi (Indonesia) which were selected using probability sampling based on cluster sampling. Respondents from different locations can be divided into each cluster [1], for example, schools that are categorized as disaster-prone area 1 because of the location that is considered safer and predicted to not get too much damage from the eruption. The use of cluster sampling in this research is according to Adhikari et al. [2] and Pilli-Sihvola et al. [3] that each individual has different skills in adaptation according to the area.

During the Covid-19 pandemic, all schools in Indonesia do online teaching and learning process so the questionnaires were also done using Google Form. The questionnaires were given to the respondents who met the criteria made by the researcher. The questionnaire aims to find out students’ preparedness in disaster adaptation based on the indicators of behavioral and psychological adaptation developed by Oakes et al. [4]. Questions related to behavioral adaptation are based on the instrument developed by Al-Amin et al. [5] and Zulch [6], while questions related to psychological adaptation are based on instruments developed by Zsido et al. [7] and Zulch [6]. 26 questions were presented using a Likert scale 1–5 points from ‘strongly disagree’ to ‘strongly agree’. The responses from the questionnaire were 364 in total and all responses successfully met the criteria for statistics analysis. The characteristics of students’ demography were analyzed using descriptive statistical analysis with one way ANOVA analysis technique to find out the relations and influences of demography characteristics to behavioral and psychological adaptation skills.

## Ethics Statement

Informed consent was obtained from all respondents. The research was done with voluntary agreement from the respondents/students. Aside from that, researcher also made agreements with the parents, teachers, and the headmaster to help the research process to run smoothly and in accordance with existing ethics. The research ethics committee of the Universiti Pendidikan Sultan Idris provided ethical approval with reference number 2021-0300-01.

## CRediT authorship contribution statement

**Siti Hadiyati Nur Hafida:** Conceptualization, Methodology, Investigation, Writing – review & editing. **Nor Kalsum Mohd Isa:** Conceptualization, Resources, Writing – review & editing. **Moh Hairy Ibrahim:** Conceptualization, Resources, Writing – original draft. **:** Resources, Writing – original draft. **Muhamad Toyib:** Formal analysis, Resources, Validation. **Muhammad Musiyam:** Investigation, Resources.

## Declaration of Competing Interest

The authors declare that they have no known competing financial interests or personal relationships that could have appeared to influence the work reported in this paper.

## Data Availability

Data for measuring disaster adaptation among students in disaster-prone areas of Mount Merapi (Original data) (Mendeley Data).
